# Untangling the relationship between negative illness perceptions and worse quality of life in patients with advanced cancer—a study from the population-based PROFILES registry

**DOI:** 10.1007/s00520-021-06179-9

**Published:** 2021-04-23

**Authors:** Lea J. Jabbarian, Judith A. C. Rietjens, Floortje Mols, Joost Oude Groeniger, Agnes van der Heide, Ida J Korfage

**Affiliations:** 1 Department of Public Health, Erasmus MC, University Medical Center Rotterdam, P.O. Box 2040, 3000 CA Rotterdam, the Netherlands; 2 Department of Medical and Clinical Psychology, Tilburg University, Tilburg, the Netherlands; 3 Netherlands Comprehensive Cancer Organisation (IKNL), Netherlands Cancer Registry, Eindhoven, the Netherlands

**Keywords:** Anxiety, Depression, Illness perceptions, Oncology, Quality of life

## Abstract

**Purpose:**

Quality of life (QoL) is an important yet complex outcome of care in patients with advanced cancer. QoL is associated with physical and psychosocial symptoms and with patients’ illness perceptions (IPs). IPs are modifiable cognitive constructs developed to make sense of one’s illness. It is unclear how IPs influence patients’ QoL. A better understanding of this relationship can inform and direct high quality care aimed at improving patients’ QoL. We therefore investigated the mediating role of anxiety and depression in the association of IPs with QoL.

**Methods:**

Data from 377 patients with advanced cancer were used from the PROFILES registry. Patients completed measures on IPs (BIPQ), QoL (EORTC QLQ-C30), and symptoms of anxiety and depression (HADS). Mediation analyses were conducted to decompose the total effect of IPs on QoL into a direct effect and indirect effect.

**Results:**

All IPs but one (“Comprehensibility”) were negatively associated with QoL (*p*<0.001); patients with more negative IPs tended to have worse QoL. The effect was strongest for patients who felt that their illness affected their life more severely (“Consequences”), patients who were more concerned about their illness (“Concern”), and patients who thought that their illness strongly affected them emotionally (“Emotions”). Anxiety mediated 41–87% and depression mediated 39–69% of the total effect of patients’ IPs on QoL.

**Conclusion:**

Negative IPs are associated with worse QoL. Anxiety and depression mediate this association. Targeting symptoms of anxiety and depression, through the modification of IPs, has the potential to improve QoL of patients with advanced cancer.

## Introduction

Patients with advanced, incurable cancer experience an impaired quality of life (QoL) [1]. Their QoL is affected in a complex way by, among others, physical symptoms and psychological challenges [2], such as the confrontation with approaching death [3] and symptoms of anxiety and depression [4, 5]. Whereas QoL is an important outcome of care, QoL is by definition multidimensional and subjective [2] and cannot be assessed by others, such as clinicians. Understanding which factors contribute to patients’ QoL is therefore of utmost importance for the delivery of high quality care to patients with advanced cancer [6].

The so-called self-regulation model conceptualizes illness perceptions as important and well-established determinants of QoL [7, 8]. Illness perceptions are defined as cognitive constructs, developed by patients to make sense of and manage their illness experience [9, 10]. Patients can adjust their illness perceptions after receiving new information, e.g., regarding the progression of the disease, from healthcare providers, the media, friends, or family [11, 12]. Illness perceptions can be in line with patients’ actual medical situation, but they can also involve a distorted interpretation of medical facts [11]. A study among patients nearing death, including patients with advanced cancer, found a great variability in illness perceptions, indicating how differently patients perceive their illness [13]. These differences may be related to the individual’s illness, cultural factors (such as the interpretation of the patient role, as well as the cultural interpretation of the illness) and factors related to an individual’s personality [9]. Due to their modifiable nature, illness perceptions are a promising target for interventions aimed at improving patients’ experiences of their illness and thereby their QoL [8, 14, 15].

The relationship between illness perceptions and physical and psychological health has been investigated in various studies. A meta-analysis of 45 studies showed that the individual illness perceptions are associated with various outcomes of social, physical, and psychological functioning [7]. More specifically, for patients with a recent cancer diagnosis, illness perceptions predicted QoL 15 months postdiagnosis, e.g., patients who thought that their cancer diagnosis had a more serious negative consequence for, among others, their relationships and finances, later reported poorer QoL [8]. While the effects of illness perceptions on QoL have been described and are recognized [8, 14, 15], there is little insight into the mechanisms underlying this relationship. Understanding these mechanisms can guide the development of future interventions aimed at the improvement of patients’ QoL. Previous research hypothesized a mediating role of anxiety and depression, since these are associated with both illness perceptions and QoL [16, 17], and are particularly common in patients with advanced cancer [18, 19]. We therefore performed a study to clarify the relationship between illness perceptions and QoL, with symptoms of anxiety and depression as potential mediators, in patients with advanced cancer, accounting for interaction effects between the illness perceptions and the mediators.

## Materials and methods

### Participants and data collection

The data were derived from the ‘Patient Reported Outcomes Following Initial treatment and Long term Evaluation of Survivorship’ (PROFILES) registry. This registry includes data to study the physical and psychosocial impact of cancer and its treatment. PROFILES is linked to the Eindhoven Cancer Registry (ECR), which includes all patients newly diagnosed with cancer in the southern part of the Netherlands. To check whether patients are still alive, these data are merged with civil municipal registries and subsequently verified by (former) treating physicians. Patients with serious cognitive impairments or in transition to terminal care are excluded. The remaining patients are invited via mail by their (former) treating physician to participate in the PROFILES registry. Interested patients can provide informed consent and complete the questionnaires via a secure website, or on paper. Patients receive questionnaires between one and four times a year. They must be able to read and write Dutch and complete a self-report questionnaire without extensive assistance. The rationale and design of PROFILES have been described elsewhere [20], data and detailed information can be found at www.profilesregistry.nl. Ethical approval for the data collection was obtained from local certified Medical Ethics Committees of the Maxima Medical Centre Veldhoven, the Netherlands (colorectal cancer, approval number 0822), the certified Medical Ethics Committee of the Maxima Medical Centre, the Netherlands ((non)Hodgkin lymphoma) and deemed exempt from full review and approval by the Research Ethics Committee Maxima Medical Centre, Veldhoven, the Netherlands (thyroid cancer). Informed consent was obtained from all individual participants included in the study. We used data from adult patients diagnosed with stage IV (non)Hodgkin lymphoma, stage IV colorectal cancer, or stage IV thyroid cancer, without cognitive impairments (*n*=377).

### Measures

#### Sociodemographic and clinical characteristics

The PROFILES registry includes the patient sociodemographic characteristics gender, age at the time of survey and at the time of diagnosis (automatically divided into ≤40 or >40 years), and time passed since the diagnosis (<2 or ≥2 years). Socioeconomic status was assessed using an indicator developed by Statistics Netherlands, based on the postal code of the residential address of the patient [21]. The registry includes the clinical characteristic tumor subtype. Patients completed the Self-administered Comorbidity Questionnaire [22].

#### Illness perceptions

The Brief Illness Perception Questionnaire (BIPQ) [23] is frequently used in cancer populations[24] and has good psychometric properties [25]. The BIPQ consists of eight items, each addressing a specific illness perception that is scored on a ten-point scale [23]:

Consequences: “How much does your illness affect your life?”

*(0—“No affect at all” to 10—“Severely affects my life”)*

Timeline: “How long do you think your illness will continue?”

*(0—“A very short time” to 10—“Forever”)*

Personal control: “How much control do you feel you have over your illness”?

*(0—“Absolutely no control” to 10—“Extreme amount of control”)*

Treatment control: “How much do you think your treatment can help your illness?”

*(0—“Not at all” to 10—“Extremely helpful”)*

Identity: “How much symptoms do you experience from your illness?”

*(0—“No symptoms at all” to 10—“Many severe symptoms”)*

Concerns:“How concerned are you about your illness?”

*(0—“Not at all concerned” to 10—“Extremely concerned”)*

Emotions: “How much does your illness affect you emotionally?”

*(0—“Not at all affected emotionally” to 10—“Extremely affected emotionally”)*

Comprehensibility: “How well do you understand your illness?”

*(0“Don’t understand at all” to 10—“Understand very clearly”)*

For the statistical analyses, we recoded the responses of three items (personal control, treatment control, and comprehensibility) to be in the same direction as the other items. Higher scores imply more negative illness perceptions (e.g., experiencing more symptoms due to the illness or being more concerned about the illness).

#### Health-related quality of life

The European Organisation for Research and Treatment of Cancer (EORTC) Quality of Life Questionnaire Core 30 (QLQ-C30; version 3.0) is an often used, validated 30-item self-reported questionnaire that contains five functional scales, three symptom scales, and six single items [26]. We calculated the recently developed QLQ-C30 summary score (range 0–100) [27]. A higher score indicates better QoL.

#### Symptoms of anxiety and depression

The Hospital Anxiety and Depression Scale (HADS) is a widely used self-reported questionnaire that measures levels of anxiety (HADS-A: seven items) and depression (HADS-D: seven items) of patients during the past week [28]. The HADS has shown good psychometric properties in various samples and settings [29]. The items are scored on a four-point Likert-scale (range total score for each subscale 0–21). A score of 8 or higher on the subscales (HADS-A and HADS-D) indicates mild to severe symptoms of anxiety or depression [29].

### Statistical analyses

Pearson correlation analyses were used to examine bivariate associations of illness perceptions, with anxiety and depression and QoL. From the original PROFILES registry, we selected the 377 patients who were diagnosed with advanced cancer. We conducted the mediation analyses with complete cases. Missing data varied from 0% for gender to 28% for comorbid conditions (Tables 1 and 2). Among the 377 patients in the total sample, 216 (57%) to 224 (59%), depending on the exposure, provided full information on the exposure (illness perceptions), mediator (anxiety or depression), outcome variable (QoL), and confounders (tumor subtype, gender, age at time of diagnosis (≤40 or >40 years), time passed since diagnosis (<2 or ≥2 years), socioeconomic status (low, medium, high, living in care institutions), and the number of comorbidities (none, 1, ≥2).

**Table 1:** Sociodemographic and clinical characteristics (*n*=377)

	No. (%)
Gender	
Male	227 (60.2)
Female	150 (39.8)
Age at time of survey	
≤ 40 years	16 (4.6)
> 40 years	334 (95.4)
Tumor subtype	
Non-Hodgkin lymphoma	52 (13.8)
Hodgkin lymphoma	192 (50.9)
Colorectal cancer	114 (30.2)
Thyroid cancer	19 (5.0)
Age at time of diagnosis	
≤ 40 years	29 (8.3)
> 40 years	322 (91.7)
Years since diagnosis	
< 2 years	77 (20.5)
≥ 2 years	299 (79.5)
Comorbid conditions	
0	95 (35.2)
1	78 (28.9)
≥2	97 (35.9)
Socioeconomic status	
Low	86 (25.1)
Middle	131 (38.2)
High	123 (35.9)
Living in a care institution	3 (0.9)

*Missings*: age at survey *n*=27, age at diagnosis *n*=26, years since diagnosis *n*=1, comorbidity *n*=107, socioeconomic status *n*=34

**Table 2:** Quality of life, illness perceptions, anxiety and depression: summary scores and correlations

	Mean (SD)	Pearson’s correlation coefficients
Quality of life (EORTC QLQ-C30)		
Quality of life	83.11 (15.70)	1.00
Illness perceptions (BIPQ)		
Consequences	4.97 (2.64)	−.49*
Timeline	6.94 (3.41)	−.17**
Personal control	5.82 (3.13)	−.21**
Treatment control	3.77 (2.61)	−.34**
Identity	4.47 (2.70)	−.55**
Concerns	4.97 (2.76)	−.17**
Emotions	4.21 (2.59)	−.46**
Comprehensibility	3.89 (2.71)	−.05
Anxiety and depression (HADS)		
Anxiety	5.10 (4.07)	−.63**
Depression	4.86 (3.98)	−.68**

*Missings*: quality of life *n*=8, consequences *n*=62, timeline *n*=54, personal control *n*=46, treatment *n*=51, identity *n*=45, concerns *n*=41, emotions *n*=43, comprehensibility *n*=40, anxiety *n*=10, depression *n*=11

*Abbreviations*: *SD*, standard deviation; *EORTC*, European Organisation for Research and Treatment, *QLQ-C30*, Quality of Life Questionnaire Core 30; *BIPQ*, Brief Illness Perception Questionnaire; *HADS*, Hospital Anxiety and Depression Scale

*p<0.05. **p<0.01

**Fig. 1:**
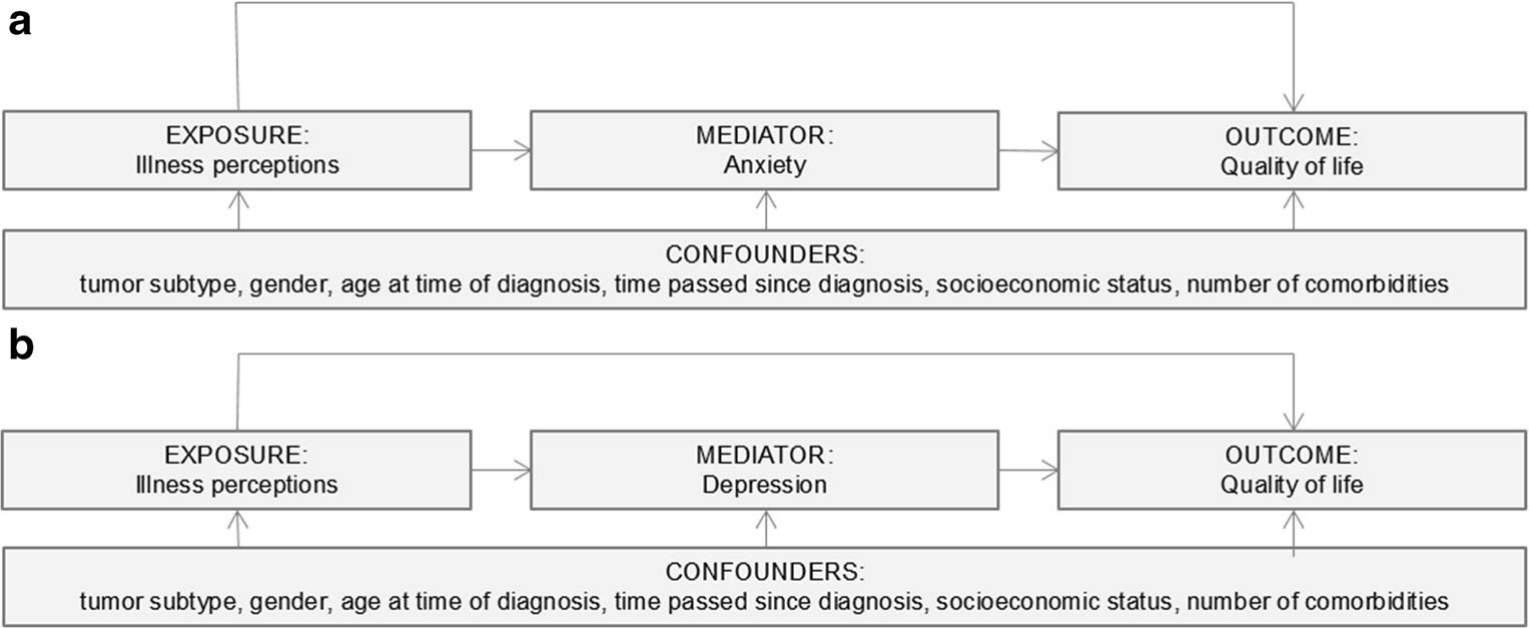
Mediation model depicting the association of illness perceptions with quality of life mediated by **a** anxiety and **b** depression

The aim of this study was to estimate how much of the observed associations of illness perceptions (exposure variables) with QoL (outcome variable) could be explained by anxiety or depression (mediators). Figure 1a and b depicts the hypothesized associations. The analyses were controlled for confounders, which were patient characteristics that, based on literature [30] and a priori assumptions, were suspected to have an impact on illness perceptions and QoL: tumor subtype, gender, age at time of diagnosis (≤40 or >40 years), time passed since diagnosis (<2 or ≥2 years), socioeconomic status (low, medium, high, living in care institutions), and the number of comorbidities (none, 1, ≥2). We found interaction effects between half of the illness perceptions and anxiety and depression on QoL. In the presence of interaction effects between exposure and mediator, traditional mediation methods such as the commonly used Baron and Kenny method, will generate invalid mediation effects [31, 32]. We therefore used a novel approach as described by Valeri and VanderWeele, which allows for exposure–mediator interactions [32]. A detailed description of this method is included in Box 1.

Box 1 Mediation analysis by Valeri and VanderWeele

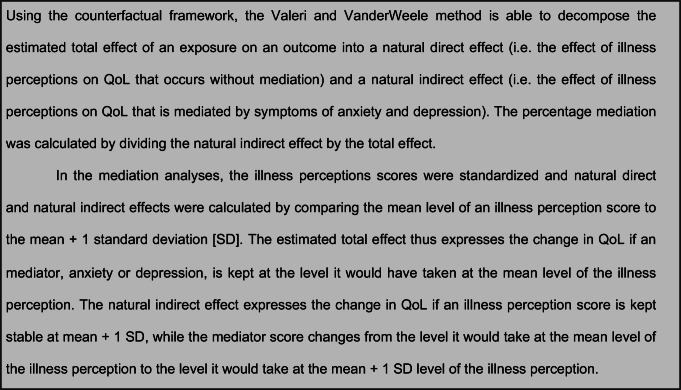



Using the counterfactual framework, the Valeri and VanderWeele method is able to decompose the estimated total effect of an exposure on an outcome into a natural direct effect (i.e., the effect of illness perceptions on QoL that occurs without mediation) and a natural indirect effect (i.e., the effect of illness perceptions on QoL that is mediated by symptoms of anxiety and depression). The percentage mediation was calculated by dividing the natural indirect effect by the total effect.

In the mediation analyses, the illness perceptions scores were standardized and natural direct and natural indirect effects were calculated by comparing the mean level of an illness perception score to the mean + 1 standard deviation [SD]. The estimated total effect thus expresses the change in QoL if an illness perception score increases from the mean to the mean + 1 SD. The natural direct effect expresses the change in QoL if an illness perception score increases from the mean to the mean + 1 SD, while the

Analyses were performed using SPSS version 21. The mediation analyses were performed using Stata version 13 with the package ‘Paramed’. *p* values <0.05 were considered to indicate statistically significant associations. 95% confidence intervals were automatically generated by the package ‘Paramed’ (based on the delta method) around the estimated total effect, natural direct effect, and natural indirect effect.

## Results

### Patient sample

The majority of patients in our sample (*n*=377) were male (60%), older than 40 years at diagnosis (92%), and diagnosed with cancer two or more years prior to participation in the study (80%, Table 1). Two or more comorbid conditions were reported by 36% of patients.

The mean summary score of the QLQ-C30 was 83.1 (SD 15.7, Table 2). Mean scores on the BIPQ are presented in Table 2. Mild to severe symptoms of anxiety were reported by 26% of patients and 25% of patients reported mild to severe symptoms of depression. All but one (“Comprehensibility”) of the illness perceptions were negatively and significantly associated with QoL (*p*<0.001), indicating that negative illness perceptions were associated with worse QoL (Table 2).

### Mediation analysis

#### Anxiety as a mediator of the association of illness perceptions with quality of life

Having more negative illness perceptions was associated with more symptoms of anxiety and having more symptoms of anxiety was associated with worse QoL. The total effect on QoL was largest for the illness perceptions “Consequences” (perceived effects and outcome of the illness on a patient’s life), “Identity” (experience of symptoms due to the illness), “Concerns” (extent to which the patient is concerned about the illness) and *“*Emotions” (emotional impact of the illness). A total of 41 to 87% of the total effect of the different illness perceptions was mediated by anxiety (Table 3). The mediating effect of anxiety was strongest for the illness perception “Emotions”. The total effect of the illness perception “Timeline” (how long the patient believes that the illness will last) on QoL, which was limited, was to a relatively large extent (84%) mediated by anxiety.

**Table 3:** Illness perceptions and quality of life: natural direct effect and indirect effect mediated by anxiety

	Total effect			Natural direct effect			Natural indirect effect			Percentage mediation
	Estimate	95%CI	*p*	Estimate	95%CI	*p*	Estimate	95%CI	*p*	%
Illness perceptions										
(1) Consequences (*n*=216)	−8.65	−1.74, −6.57	.000	−4.60	−6.44, −2.76	.000	−4.05	−5.52, −2.59	.000	47%
(2) Timeline (*n*=216)	−1.80	−3.87, .27	.088	−.28	−2.01, 1.44	.747	−1.52	−2.66, −.37	.009	84%
(3) Personal control (*n*=223)	−3.12	−5.18, −1.05	.003	−1.04	−2.73, .65	.228	−2.08	−3.32, −.83	.001	67%
(4) Treatment control (*n*=219)	−5.48	−7.53, −3.43	.000	−2.91	−4.63, −1.2	.001	−2.56	−3.89, −1.24	.000	47%
(5) Identity (*n*=220)	−7.81	−9.71, −5.92	.000	−4.61	−6.32, −2.89	.000	−3.21	−4.48, −1.94	.000	41%
(6) Concerns (*n*=223)	−7.03	−9.1, −4.96	.000	−1.95	−4, .09	.062	−5.08	−6.73, −3.44	.000	72%
(7) Emotions (*n*=224)	−6.43	−8.29, −4.57	.000	−.86	−3.09, 1.36	.446	−5.57	−7.34, −3.79	.000	87%
(8) Comprehensibility^a^ (*n*=222)	−.37	−2.32, 1.58	.708	.80	−.85, 2.44	.344	−1.17	−2.3, −.04	.042	

^a^Comprehensibility affects quality of life via opposing direct and indirect effects. This makes calculating the mediated effect nonsensical

#### Depression as a mediator of the association of illness perceptions with quality of life

Having more negative illness perceptions was associated with more symptoms of depression, which, in turn, was associated with worse QoL. Depression mediated 39 to 69% of the effect of illness perceptions on QoL (Table 4). The mediating effects of depression were strongest for the illness perceptions “Emotions”, “Concerns”, and “Consequences”. The limited total effect of the illness perception “Timeline” on QoL was to relatively large extent (69%) mediated by depression. In general, the mediating effects of depression were somewhat weaker than the mediating effects of anxiety.

**Table 4:** Illness perceptions and quality of life: natural direct effect and indirect effect mediated by depression

	Total effect			Natural direct effect			Natural indirect effect			Percentage mediation
	Estimate	95%CI	*p*	Estimate	95%CI	*p*	Estimate	95%CI	*p*	%
Illness perceptions										
(1) Consequences (*n*=216)	−8.02	−1.01, −6.04	.000	−4.19	−5.95, −2.43	.000	−3.83	−5.25, −2.41	.000	48%
(2) Timeline (*n*=216)	−2.08	−4.16, .01	.051	−.64	−2.29, 1.01	.447	−1.44	−2.71, −.16	.028	69%
(3) Personal control (*n*=223)	−2.98	−4.98, −.98	.003	−1.27	−2.86, .33	.119	−1.71	−2.97, −.46	.007	57%
(4) Treatment control (*n*=219)	−5.45	−7.48, −3.41	.000	−2.68	−4.35, −1.01	.002	−2.77	−4.14, −1.39	.000	51%
(5) Identity (*n*=220)	−7.70	−9.59, −5.81	.000	−4.71	−6.31, −3.11	.000	−2.99	−4.28, −1.71	.000	39%
(6) Concerns (*n*=223)	−6.81	−8.8, −4.81	.000	−2.88	−4.63, −1.13	.001	−3.93	−5.36, −2.49	.000	58%
(7) Emotions (*n*=224)	−6.72	−8.62, −4.83	.000	−2.79	−4.48, −1.1	.001	−3.94	−5.33, −2.54	.000	59%
(8) Comprehensibility^a^ (*n*=222)	−.35	−2.28, 1.58	.723	.97	−.63, 2.56	.235	−1.32	−2.49, −.14	.028	

^a^Comprehensibility effects quality of life via opposing direct and indirect effects. This makes calculating the mediated effect nonsensical

## Discussion

This study explored the mediating role of anxiety and depression in the association of illness perceptions with QoL in a large sample of patients with advanced cancer. We were able to confirm prior findings that having more negative illness perceptions (e.g., experiencing more symptoms due to the illness, being more concerned about the illness) is associated with worse QoL. Our study adds that this association is substantially mediated by symptoms of anxiety or depression.

It is not surprising that the total effect of the illness perception “Emotions” (emotional impact of the illness) on QoL was the largest and to a relatively large extent mediated by symptoms of anxiety and depression, considering that this item measures the emotional impact of the illness on the patient. In accordance with previous research among patients treated for breast cancer [33], we found that patients who feel that their illness affects their life more severely (“Consequences”) and who experience many symptoms from their illness (“Identity”) have a considerable worse QoL. Our findings add that nearly half of that association was mediated by symptoms of anxiety or depression. Patients scoring high on “Identity” tend to attribute commonly occurring symptoms (such as a headache) to their illness, even if no such association exists [34]. This applies in particular to patients with advanced cancer who have to deal with uncertainty about the extent to which their life expectancy is limited and who tend to interpret symptoms as signs of potential progression of their illness [35, 36]. We now know that over-interpretation of symptoms may lead to symptoms of anxiety and depression, which in turn impairs QoL.

Patients had the highest average score on the illness perception “Timeline”, meaning that they believed that their illness would last “forever”. Previous research has shown that “Timeline” scores were skewed toward the upper extreme in patients with advanced cancer, which suggests awareness of the incurable nature of their illness [13]. “Timeline” scores were only to a limited extent associated with QoL. This association however was to a large extent mediated by symptoms of anxiety and depression, meaning that being aware of the limited life expectancy does not have a strong direct effect on QoL itself, but mainly impacts QoL negatively through the strong experience of symptoms of anxiety and depression.

Understanding how patients with advanced cancer make sense of their diagnosis and addressing these illness perceptions is a promising approach when supporting patients with symptoms of anxiety or depression, and can thus be a way of improving the QoL of patients with advanced cancer. Since the prevalence of symptoms of anxiety and depression is higher in patients with advanced cancer than in colorectal cancer survivors, and even higher in comparison to the normative population [37], patients are in clear need of support. Our findings emphasize the importance of raising awareness for patients’ illness perceptions [38, 39], especially since previous research found that healthcare providers’ understanding of the illness perceptions of their patients was relatively poor [40], also with regard to important topics such as prognosis [41]. The recent consensus guideline of the American Society of Clinical Oncology on patient-clinician communication highlights the importance of (improved) health care communication and its positive impact on many objective and subjective health outcomes [38]. Incorporating the discussion of illness perceptions may play an important role in the patient-clinician communication and in meeting patients’ information needs [30]. Additionally, previous research indicated the usefulness of targeting illness perceptions as a way to improve health outcomes [42]. Patients who were recovering from a myocardial infarction found a brief intervention on altering illness perceptions to be effective in improving functional outcomes [42]. Moreover, a recent study with patients with unruptured intracranial aneurysm found that cognitive behavioral therapy reduces feelings of anxiety and improves illness perceptions [43]. Given that cognitive behavioral therapy has been proven effective in the treatment of mood disorders in patients with cancer, it would be worthwhile to investigate its application in patients with (advanced) cancer [44].

The main strengths of this study lie in the use of a relatively large dataset of patients with advanced cancer, a unique and vulnerable group of patients that is rarely investigated, and the use of recently developed, advanced mediation analysis techniques that allow for the decomposition of total effects into natural direct and indirect effects, while accounting for exposure–mediator interactions.

Some limitations need to be considered when interpreting the findings. Although previous research and theoretical models suggest a strong temporal sequence, with illness perceptions preceding symptoms of anxiety and depression [17], this study cannot draw causal conclusions due to its cross-sectional study design. Second, to interpret the observed direct and indirect effects, one needs to assume that there are no unmeasured confounders of the exposure–mediator relationship, the mediator-outcome relationship and the exposure–outcome relationship [32]. Although we did adjust for several potential confounders, we cannot exclude the possibility that unmeasured confounders may have impacted the results. Third, we performed a complete case analysis on the subset of patients with full information on the exposure, mediator, outcome variables and confounders. While this method is widely applied to treat missing data, it may lead to biased results if the data are not missing completely at random [45].

In conclusion, our study indicates that negative illness perceptions are associated with worse QoL in patients with advanced cancer. Symptoms of anxiety and depression substantially mediate this association. Applying this knowledge to patient-clinician interactions may improve its quality and ultimately the QoL of patients with advanced cancer. Further prospective research is needed to confirm these findings and extend the exploration of hidden mechanisms behind the relationship between illness perceptions and QoL, by looking at the role of e.g., personality traits and coping styles, physical factors such as comorbidities and different types and stages of cancer, health literacy, cultural factors, or the quality of patient–clinician interactions. QoL and symptoms of anxiety and depression in patients with advanced cancer may be improved by addressing illness perceptions during medical consultations.

## Data Availability

Data and detailed information can be found at www.profilesregistry.nl.
